# Blackcurrant Extract with Phytoestrogen Activity Alleviates Hair Loss in Ovariectomized Rats

**DOI:** 10.3390/molecules24071272

**Published:** 2019-04-01

**Authors:** Naoki Nanashima, Kayo Horie

**Affiliations:** Department of Bioscience and Laboratory Medicine, Hirosaki University Graduate School of Health Sciences, 66-1 Hon-cho, Hirosaki, Aomori 036-8564, Japan; k-horie@hirosaki-u.ac.jp

**Keywords:** ancocyanin, blackcurrant, female pattern hair loss, ovariectomized rat, phytoestrogen

## Abstract

Ancocyanin-rich blackcurrant extract (BCE) has phytoestrogen activity; however, its effect on hair follicles is unknown. Additionally, hair loss is known to occur during menopause in women owing to decreased estrogen secretion. This study examined whether BCE alleviated female pattern hair loss using a rat model. RNA was extracted and analyzed using a microarray and ingenuity pathway analysis. A quantitative polymerase chain reaction revealed that 1 μg/mL BCE altered many genes downstream of beta-estradiol in human hair dermal papilla cells. Additionally, the expression of the hair follicle stem cell marker keratin 19 was greatly enhanced. In a menopause model, ovariectomized rats were fed a diet containing 3% BCE for three months. An analysis of the number of hair shafts revealed that BCE increased the number of hairs by 0.5 hairs/follicular unit. Moreover, immunostaining revealed that the expression of Ki67 also increased by 19%. Furthermore, fluorescent immunostaining showed that the expression of other stem cell markers, including keratin 15, CD34, and keratin 19, was induced in rat hair follicular cells. In conclusion, these findings suggest that BCE has phytoestrogen activity in hair follicles and contributes to the alleviation of hair loss in a menopausal model in rats.

## 1. Introduction

Blackcurrant (*Ribes nigrum* L.) contains high levels of anthocyanins, including cyanidin-3-glucoside, cyanidin-3-rutinoside, delphinidin-3-glucoside, and delphinidin-3-rutinoside [1]. These anthocyanins are reported to have some health benefits, such as in the prevention of breast cancer and reduction of inflammation and obesity [2,3,4]. Furthermore, blackcurrant contains various bioactive compounds and has been traditionally used in the treatment of various conditions, including rheumatic disease [5].

Phytoestrogens, including isoflavones, lignans, coumestans, and flavonoids, which are found in many foods, are chemically diverse plant compounds that exert estrogenic effects in animals [6,7,8,9]. Recent studies have reported that blackcurrant extract (BCE) and anthocyanins derived from blackcurrant act as phytoestrogens by signaling through both estrogen receptor (ER)α and ERβ [10,11]. 

Estrogens affect the functions of various organs and tissues, including blood vessels, bones, brain, skin, and hair, and participate in the mechanisms underlying several diseases, such as metabolic syndrome [12,13,14,15]. The function of women’s ovaries decreases gradually beginning in their late 20s. Moreover, estrogen secretion decreases sharply at around 40 years of age; this decrease is associated with menopausal disorder, which involves symptoms such as thinning hair, hair loss, hot flashes, headaches, dizziness, palpitations, and malaise.

To alleviate menopausal symptoms, postmenopausal women may take hormone replacement therapy. However, it is necessary to consider the risk of venous thrombosis and breast cancer when using estrogen preparations. In contrast, phytoestrogens have not been reported to be associated with these risks and are therefore considered important substitutes for estrogen preparations [16,17]. In female pattern hair loss (FPHL), such as that occurring during menopause, the number of hairs decrease at the head top in the center, which makes the hair thin [18]. There are few changes in the crown and hairline. Ovariectomy decreases the secretion of estrogen from the ovary, therefore ovariectomized (OVX) rodents are widely used as a menopausal model [19]. Furthermore, OVX mice have been reported to be useful animal models of FPHL [20,21]. 

The hair follicle consists of epithelial and mesenchymal tissues. Hair follicle stem cells are involved in the production of hair and supply the necessary cells to make hair by division and differentiation. Keratin (K) 19, K15, and cluster of differentiation (CD) 34 are hair follicle stem cell markers [22,23,24]. K19 is expressed in the epidermis of the skin, the bulge region of the hair follicle, and the outermost layer of the outer root sheath [25,26,27]. CD34 and K15 are localized on the hair bulge region, and K15 is also localized on the hair germ [28,29,30]. Hair follicle dermal papilla cells (HFDPCs) are important cells present at the tip of the hair follicle and take up nutrients from capillaries to grow hair [31,32]. 

Previous studies reported that BCE and blackcurrant anthocyanins improved dermal health and increased the production of collagen, elastin, and hyaluronic acid in OVX rats [33]. However, the effects of phytoestrogen on hair follicles are still unknown. Accordingly, this study aimed to investigate the beneficial effects of BCE on FPHL in OVX rats as a menopausal model. 

## 2. Results and Discussion

### 2.1. Gene Expression Profiling of HFDPCs Exposed to BCE

Previous studies revealed that 1.0 μg/mL BCE had phytoestrogen effects [10,11]. However, the effects of BCE in scalp HFDPCs were still unclear. Therefore, this study first compared gene expression in HFDPCs derived from the temples of Caucasian middle-aged women before and after exposure to BCE (1.0 μg/mL) using microarrays. Ingenuity pathway analysis (IPA) was performed to investigate the biological functional relationships between sets of genes showing altered expression levels. Several predicted upstream regulators were detected; however, only beta-estradiol and estrogen receptor had z-scores of greater than 2.0 (z-score = 3.07 and 2.14, respectively; Table 1). The HFDPCs used in this study did not express ERβ, whereas ERα mRNA was expressed robustly (data not shown). This suggests that the observed upregulation of genes downstream of beta-estradiol and the estrogen receptor by BCE was due to ERα activation, which is consistent with a previous study using skin fibroblast [33].

The z-scores of peroxisome proliferator-activated receptor γ (PPARG) and glucocorticoid were both 2.2. Thus, these results showed that BCE acted similarly to beta-estradiol in HFDPCs and stimulated not only estrogen signaling but also PPARG and glucocorticoid signaling. PPARG has important functions in human hair follicle epithelial stem cells [34], and PPARG gene knockout targeted to stem cells of the follicular bulge caused scarring alopecia in mice [35]. Glucocorticoids affect hair growth initiation [36], and glucocorticoid receptors are involved in epidermal homeostasis and hair follicle differentiation [37]. Thus, BCE appeared to stimulate hair growth-related signaling in HFDPCs.

**Figure 1:**
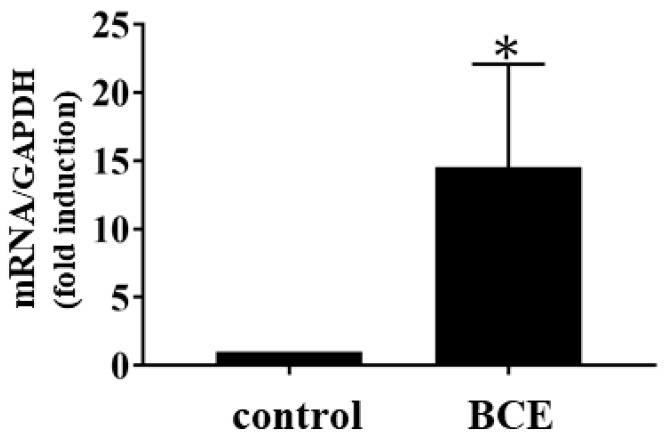
Effects of BCE on *K19* mRNA levels. HFDPCs were treated with 1.0 μg/mL BCE for 24 h, and mRNA levels were quantified by quantitative polymerase chain reaction (qPCR). Data represent the means ± standard deviations for at least three independent experiments. * *p* < 0.05 versus untreated control cells.

In addition, the expression of genes downstream of beta-estradiol was markedly affected by BCE treatment (Table 2). These results indicate that BCE significantly alters the expression levels of genes involved in pathways related to estrogen signaling. Whole transcript microarray analysis of HFDPCs exposed to BCE (1.0 μg/mL) showed that K19 expression demonstrated the most dramatic increase (Table 2). To confirm whether BCE affected the expression of K19, HFDPCs were incubated with 1.0 μg/mL BCE for 24 h. Quantitative polymerase chain reaction (qPCR) analysis showed that BCE induced a 14.6-fold increase (*p* < 0.05) in *K19* mRNA levels (Figure 1). As K19 is a marker for hair follicle stem cells, these findings suggest that BCE enhances stemness in HFDPCs. *Homeobox* (*HOX*) *C6* and *HOXC4* expression levels also increased with BCE treatment (Table 2); these genes are known to be sufficient to reprogram mesenchymal dermal papilla cell [38]. 

Thus, because BCE increased the expression of hair growth and hair follicle stem-related genes in HFDPCs, these findings suggest that BCE has estrogenic activity in HFDPCs and could contribute to the regulation of many hair growth-related genes.

### 2.2. BCE Alleviated Hair Loss in OVX Rats

**Figure 2:**
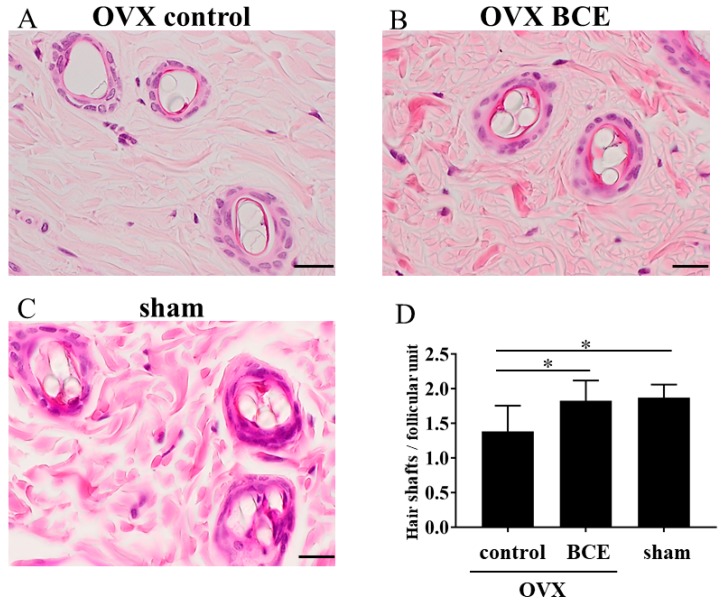
Number of hair shafts per follicular unit. (**A**) *n* = 7, ovariectomized (OVX) control; (**B**) *n* = 6, OVX BCE; (**C**) *n* = 6, sham. Scale bar = 20 μm. (**D**) The skin tissues of six to seven rats were evaluated to determine hair shaft counts in each of the three fields. Data represent the means ± standard deviations. * *p* < 0.05.

OVX rats were used as menopausal model animals and changes were examined in FPHL in response to BCE. OVX rats were given 3% BCE (OVX BCE), skin samples were taken after three months, and the number of hair shafts per follicular unit was counted. Compared with the OVX control (1.4 ± 0.1), the number of hair shafts per follicular unit increased slightly (*p* < 0.05) in the OVX BCE and sham groups (1.8 ± 0.1 and 1.9 ± 0.1, respectively; Figure 2A–D). Thus, the number of hair shafts per follicular unit in BCE-treated OVX rats increased by approximately 0.5 shafts/follicular unit, similar to that in sham rats. This result suggests that these rats can be used as a model of FPHL, and that BCE has some effect on alleviating menopausal hair loss.

### 2.3. BCE Enhanced the Proliferation of Hair Follicle Cells

**Figure 3:**
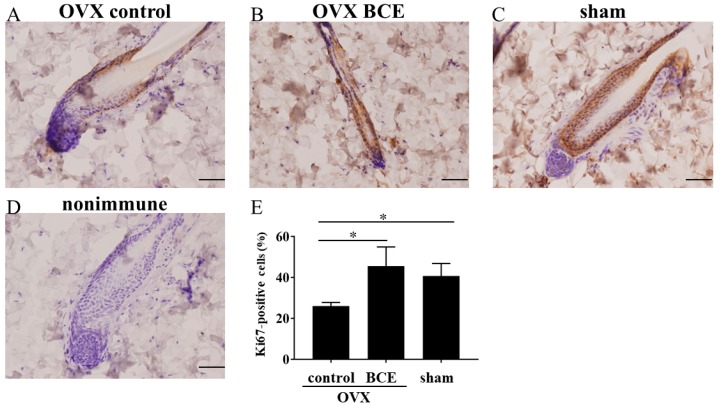
Evaluation of cell proliferation by expression of Ki67. Skin from each rat was stained with anti-Ki67 antibodies. As a negative control, nonimmune γ-globulin was used instead of the antibody. (**A**) *n* = 7, OVX control; (**B**) *n* = 6, OVX BCE; (**C**) *n* = 6, sham. (**D**) As a negative control, nonimmune γ-globulin was used instead of the antibody. Scale bar = 100 μm. (**E**) Ki67-positive cells in the skin tissues were counted for hair follicles in each of the three fields. Data represent the means ± standard deviations. * *p* < 0.05.

Subsequently, the study investigated the expression of Ki67, a cell proliferation marker, by immunostaining to determine whether the intake of 3% BCE induced cell proliferation in the hair follicle cells of OVX rats. For Ki67-positive cells, the percentage of proliferative cells in the OVX control was 26.1% ± 1.7%, whereas those in the OVX BCE and sham groups were 45.6% ± 3.1% and 40.7% ± 6.1%, respectively (*p* < 0.05, Figure 3). Some phytochemicals are known to increase the expression of Ki67 in hair follicles and promote hair growth [39]. Indeed, this study also found that BCE induced Ki67 expression, suggesting that BCE induces hair follicle cell proliferation and promotes hair growth in OVX rats. Moreover, these findings support the view that FPHL is caused by the proliferation of cells within hair follicles owing to decreased estrogen. Furthermore, the phytoestrogen effects of BCE appeared to alleviate the decrease in Ki67 expression.

### 2.4. Effects of BCE on the Expression of the Hair Follicle Stem Cell Marker K19 in OVX Rats

**Figure 4:**
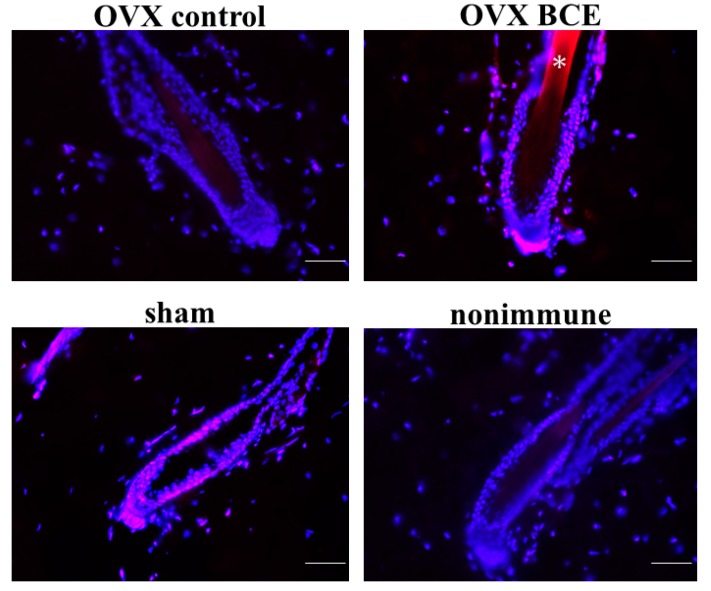
Induction of K19 expression in the hair follicles by BCE. Skin from rats was stained with anti-K19 antibodies (red signals). Nuclei were visualized using 4′,6-diamidino-2-phenylindole (blue). As a negative control, nonimmune γ-globulin was used instead of the antibody. *Autofluorescence from the hair shaft. Scale bar = 100 μm.

As BCE was found to induce K19 in HFDPCs, the study then investigated whether K19 expression was increased in OVX rats. The expression of K19 was low in the OVX control, but increased in the bulge region in the OVX BCE and sham groups (Figure 4). K19 expression is known to correlate significantly with Ki67 expression [40]. Therefore, the results suggest that BCE induced the expression of K19 not only in vitro but also in vivo, contributing to the maintenance of stem cells and cell proliferation.

### 2.5. Effects of BCE on the Expression of the Hair Follicle Stem Cell Markers K15 and CD34 in OVX Rats

**Figure 5:**
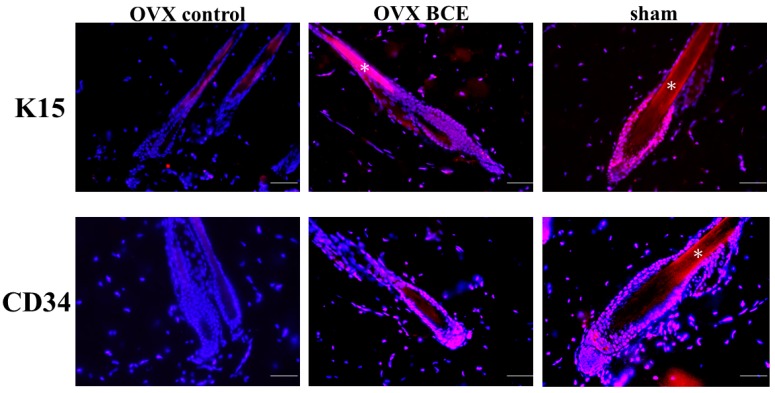
Induction of K15 and CD34 expression in hair follicles by BCE. Skin from treated rats was stained with anti-K15 antibodies (red signals) or anti-CD34 antibodies (red signals). The nuclei were visualized using 4′,6-diamidino-2-phenylindole. * Autofluorescence from the hair shaft. Scale bar = 100 μm.

The study then examined the expression of hair stem cell markers, including K15 and CD34. Using anti-K15 antibodies, the bulge region in the hair follicles of the OVX BCE and sham groups showed a positive reaction, but the staining of hair follicles from the OXV control group was negative (Figure 5). Staining with anti-CD34 antibodies revealed a positive reaction in the bulge region (Figure 5). These positive cells were putative hair follicle stem cells. The low expression of K19, K15, and CD34 in OVX rats may have decreased the stemness of hair follicle stem cells, suggesting the involvement of stemness in FPHL. Although little is known about the stemness of hair follicles, one report showed that the antibiotic ciprofloxacin enhances the stemness of human hair papilla cells [41]. Thus, further studies are needed to determine whether BCE increases the stemness of hair follicles. Furthermore, BCE may activate hair follicle stem cells and contribute to an improvement of stemness. 

The PPARG modulator GMG-43AC stimulates K15 and K19 [42]. As BCE enhanced PPARG signaling (Table 1), BCE may not only affect phytoestrogen activity but may also modulate PPARG in HFDPCs and hair follicles. Previous studies revealed that blackcurrant anthocyanins stimulated the expression of genes encoding extracellular matrix (ECM) components and the tissue inhibitor of matrix metalloproteinase (MMP) 3, but downregulated the ECM protein degradation enzyme MMP12 in female skin fibroblasts. Furthermore, dietary administration of 3% BCE into ovariectomized rats for three months also increased skin levels of ECM compounds [33]. The ECM acts through the Wnt pathway to regulate hair epidermal stem cell activity, which is important for the modulation of the hair follicle stem cell niche [43]. Thus, the results of previous studies and this study suggest that BCE may contribute to the alleviation of menopause-related hair loss by facilitating interactions between the ECM and hair follicle stem cells.

## 3. Materials and Methods

### 3.1. Materials and Cell Culture

BCE powder, CaNZac-35, was purchased from Koyo Mercantile Co. (Tokyo, Japan). BCE contained high concentrations of anthocyanins (38.0 g/100 g BCE) [11]. HFDPCs derived from the temples of Caucasian middle-aged women were obtained from PromoCell (Heidelberg, Germany). Cells were maintained in follicle dermal papilla cell growth medium (PromoCell). All cell culture experiments were conducted at 37 °C in a humidified incubator containing 5% CO_2_.

### 3.2. Microarray Gene Expression Profiling

HFDPCs were seeded in 21 cm^2^ culture dishes and grown until reaching confluence. The medium was then replaced with phenol red-free follicle dermal papilla cell basal medium, with or without BCE (1.0 μg/mL) for 24 h. After washing cells twice with phosphate-buffered saline (PBS), total RNA was extracted using an RNeasy mini kit (Qiagen, Hilden, Germany) according to the manufacturer’s instruction. RNA labeling and hybridization were performed using the Agilent One-Color Microarray-Based Gene Expression Analysis protocol (version 6.5, 2010; Agilent Technologies, Santa Clara, CA, USA). Briefly, 100 ng total RNA from each sample was linearly amplified and labeled with Cy3-dCTP. The resulting labeled cRNAs were purified using an RNAeasy mini kit (Qiagen). Labeled and fragmented cRNA was hybridized to a SurePrint G3 Human Gene Expression microarray (8 × 60 K version 3; Agilent Technologies). Labeling, hybridization, image scanning, and data analysis were performed at Macrogen Japan Corp. (Tokyo, Japan). The microarray dataset is accessible at http://www.ncbi.nlm.nih.gov/geo under accession code GSE 117360.

### 3.3. IPA

Genes showing a greater than 2.0-fold upregulation following exposure of HFDPCs to 1.0 μg/mL BCE were analyzed using IPA software (version 36601845) (Qiagen, Germantown, MD, USA). The z-score algorithm was utilized to reduce the possibility of false-positive results, where z scores greater than 2.0 indicated that transcript expression was significantly increased, and z scores less than −2.0 indicated that expression was significantly decreased.

### 3.4. qPCR

HFDPCs were seeded in 21 cm^2^ culture dishes and grown until reaching confluence. The medium was then replaced with phenol red-free follicle dermal papilla cell basal medium, with or without BCE (1.0 μg/mL). Cells were incubated for 24 h and washed twice with PBS. Total RNA was prepared using an RNeasy mini kit (Qiagen) according to the manufacturer’s instructions. cDNA was reverse-transcribed using PrimeScript RT Master Mix (TaKaRa, Tokyo, Japan). Levels of *K19* mRNA were quantified by qPCR using TB Green Premix Ex Taq II (Tli RNaseH Plus; TaKaRa). The PCR amplification protocol consisted of 30 s at 94 °C, 30 s at 60 °C, and 30 s at 72 °C for 40 cycles. Transcript levels were normalized to those of glyceraldehyde 3-phosphate dehydrogenase (*GAPDH*) cDNA. The primers were as follows (5′→3′): *K19*, forward CGGGACAAGATTCTTGGT and reverse CGTTGATGTCGGCCTCCA [44]; *GAPDH*, forward TGAGAACGGGAAGTCTGTCA and reverse TCTCCATGGTGGTGAAGACG. PCR specificity was checked using s melting curve analysis. All samples were analyzed in duplicate, and relative gene expression was calculated according to the 2^−ΔΔCt^ method.

### 3.5. Animals and Treatments

OVX female Sprague–Dawley and sham surgery rats (12 weeks of age) were purchased from CLEA Japan Inc. (Tokyo, Japan). The rats were housed in air-conditioned rooms with a 12 h light/dark cycle and free access to water and food at the Institute for Animal Experiments of Hirosaki University Graduate School of Medicine. Previous studies showed that 3% BCE elicited phytoestrogen effects in the rat uterus and skin [11,33]. All rats received an AIN-93M diet, with or without 3% BCE, as indicated (CLEA Japan, Inc.), and were divided into three groups (*n* = 6–7/group) as follows: (1) OVX rats treated with 3% BCE for three months (OVX BCE group), (2) OVX rats without BCE treatment (OVX control group), and (3) sham surgery rats without BCE treatment (sham group). At the end of the experiment, the animals were euthanized and the back skin was removed. The skin tissues were fixed in 10% formaldehyde and embedded in paraffin for histological examination. This experiment was approved by the Animal Research Committee of Hirosaki University (permission number: G16004) and was conducted in accordance with the rules for Animal Experimentation of Hirosaki University.

### 3.6. Enumeration of Hair Shafts per Follicular Unit and Analysis of Immunohistochemical Staining

Skin tissue sections (3 μm thick) were mounted onto silane-coated slides. The sections were deparaffinized by passing through xylene and a graded alcohol series before being stained with hematoxylin and eosin. The number of hair shafts per follicular unit was counted using a fluorescence microscope (FSX100; Olympus, Tokyo, Japan). In immunohistochemistry, tissue sections were heated in a microwave oven to 95 °C in citric acid buffer (pH 6.0) for 15 min for antigen retrieval, and then incubated with anti-Ki67 antibodies (cat. no. ab15580; Abcam, Cambridge, MA, USA; 1:100 dilution, *v*/*v*) at 4 °C overnight. The tissue sections were then incubated at room temperature for 60 min with a universal secondary antibody (Roche, Basel, Switzerland). The site for peroxidase binding was determined using DISCOVERY DAB Map Detection Kit (Roche). Sections were then counterstained with Hematoxylin II (Roche) for microscopic examination. As a negative control, nonimmune γ-globulin was used instead of the antibody. Images were captured using a fluorescence microscope (FSX100). Ki67-positive cells were counted, and the percentage was calculated relative to nuclear staining.

### 3.7. Immunofluorescence Staining

Deparaffinization and antigen retrieval was carried out as described above. Subsequently, the slides were incubated with anti-cytokeratin 19 (A-3) antibodies (cat. no. sc-376126; Santa Cruz Biotechnology, Santa Cruz, CA, USA; 1:50 dilution, *v*/*v*), anti-cytokeratin 15 (LHK15) antibodies (cat. no. sc-47697; Santa Cruz Biotechnology; 1:50 dilution, *v*/*v*), or anti-CD34 (B-6) antibodies (cat. no. sc-74499; Santa Cruz Biotechnology; 1:50 dilution, *v*/*v*) at 4 °C overnight. This was followed by incubation with Alexa Fluor 546 anti-mouse IgG secondary antibodies (cat. no. A11060; Life Technologies; dilution 1:500) for 30 min at room temperature. Nuclear staining and mounting were performed using Vectashield Mounting Medium with 4′,6-diamidino-2-phenylindole (Vector Laboratories, Burlingame, CA, USA). As a negative control, nonimmune γ-globulin was used instead of the antibody. Images were captured using a fluorescence microscope (FSX100).

### 3.8. Statistical Analysis

Results are expressed as the means ± standard deviations. Graphs were generated using Graph Pad Prism 7.0 ver. 7.03 software (Graph Pad Prism, San Diego, CA, USA). All statistical analyses were performed using BellCurve for Excel ver. 3.0 software (Social Survey Research Information, Tokyo, Japan). The qPCR results were evaluated using Student’s *t*-tests. Comparisons of the three groups were analyzed using one-way analysis of variance with Tukey–Kramer post-hoc tests, whereas comparisons of the two groups were evaluated using *t*-tests. Differences with *p* values of less than 0.05 were considered statistically significant.

## 4. Conclusions

Because BCE has phytoestrogen activity, this study investigated the effects of BCE on hair loss in OVX rats. The results showed that BCE affected the expression of many genes downstream of beta-estradiol and estrogen receptor in human HFDPCs, most notably *K19*. BCE also increased the number of hairs per hair follicle and the expression of the cell proliferation marker Ki67 in OVX rats. In addition, BCE enhanced the expression of hair follicle stem cell markers, such as K19, K15, and CD34, in OVX rats, similar to that in sham rats. These results suggest that BCE has phytoestrogen effects and enhanced stemness in the hair follicles of OVX rats. Further studies are necessary to determine whether BCE is an effective treatment for FPHL in menopausal women.

## Figures and Tables

**Table 1:** Ingenuity pathway analysis (IPA) of hair follicle dermal papilla cells (HFDPCs) treated with blackcurrant extract (BCE).

Predicted Upstream Regulator	Z-Score
Beta-estradiol	3.07
PPARG	2.2
Glucocorticoid	2.2
Estrogen receptor	2.14

**Table 2:** Effects of BCE on beta-estradiol-regulated gene expression in HFDPCs.

Gene Symbol	Gene Name	Fold Change
*KRT19*	*Keratin 19*	58.8
*HOXC6*	*Homeobox C6*	18.1
*NR4A3*	*Nuclear receptor subfamily 4 group A member 3*	9.5
*HOXC4*	*Homeobox C4*	8.7
*CDH2*	*Cadherin 2*	5.8
*HSPB8*	*Heat shock protein family B (small) member 8*	4.4
*HSD17B7*	*Hydroxysteroid 17-beta dehydrogenase 7*	2.7
*PPARGC1B*	*PPARG coactivator 1 beta*	2.6
*AGT*	*Angiotensinogen*	2.4
*NKX3-1*	*NK3 homeobox 1*	2.3
*SEMA3F*	*Semaphorin 3F*	2.2
*HTRA3*	*HtrA serine peptidase 3*	2.2
*SLC52A1*	*Solute carrier family 52 member 1*	2.2
*FASN*	*Fatty acid synthase*	2.1
*CYP1B1*	*Cytochrome P450 family 1 subfamily B member 1*	2.1
*SLC7A5*	*Solute carrier family 7 member 5*	2.1
